# STxB as an Antigen Delivery Tool for Mucosal Vaccination

**DOI:** 10.3390/toxins14030202

**Published:** 2022-03-10

**Authors:** Eric Tartour, Ludger Johannes

**Affiliations:** 1 PARCC, INSERM U970, Université de Paris, 75006 Paris, France; 2 Equipe Labellisée Ligue Contre le Cancer, 75013 Paris, France; 3 Service d’Immunologie Biologique, APHP, Hôpital Européen Georges Pompidou, 75015 Paris, France; 4 Institut Curie, Université PSL, U1143 INSERM, UMR3666 CNRS, Cellular and Chemical Biology Unit, 26 Rue d’Ulm, CEDEX 05, 75248 Paris, France

**Keywords:** glycolipid-lectin, GL-Lect, endosomal escape, cross-presentation, tissue resident memory T cells, TRM, immune checkpoint, radiotherapy, chemotherapy, cytotoxic CD8^+^ T lymphocyte

## Abstract

Immunotherapy against cancer and infectious disease holds the promise of high efficacy with minor side effects. Mucosal vaccines to protect against tumors or infections disease agents that affect the upper airways or the lung are still lacking, however. One mucosal vaccine candidate is the B-subunit of Shiga toxin, STxB. In this review, we compare STxB to other immunotherapy vectors. STxB is a non-toxic protein that binds to a glycosylated lipid, termed globotriaosylceramide (Gb3), which is preferentially expressed by dendritic cells. We review the use of STxB for the cross-presentation of tumor or viral antigens in a MHC class I-restricted manner to induce humoral immunity against these antigens in addition to polyfunctional and persistent CD4^+^ and CD8^+^ T lymphocytes capable of protecting against viral infection or tumor growth. Other literature will be summarized that documents a powerful induction of mucosal IgA and resident memory CD8^+^ T cells against mucosal tumors specifically when STxB-antigen conjugates are administered via the nasal route. It will also be pointed out how STxB-based vaccines have been shown in preclinical cancer models to synergize with other therapeutic modalities (immune checkpoint inhibitors, anti-angiogenic therapy, radiotherapy). Finally, we will discuss how molecular aspects such as low immunogenicity, cross-species conservation of Gb3 expression, and lack of toxicity contribute to the competitive positioning of STxB among the different DC targeting approaches. STxB thereby appears as an original and innovative tool for the development of mucosal vaccines in infectious diseases and cancer.

## 1. Shiga Toxin and Its Intracellular Trafficking

**Figure 1:**
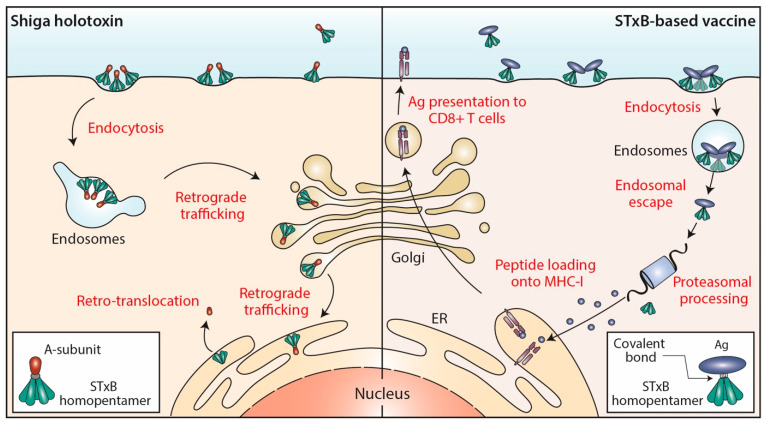
STxB trafficking into cells. Left: Shiga holotoxin molecules are composed of a STxB homopentamer (green) and a catalytic A-subunit (red), which are non-covalently associated. STxB binds to the plasma membrane of target cells via the glycosphingolipid Gb3 (not shown). STxB induces an increment of spontaneous curvature, which upon membrane-mediated clustering of several toxin molecules leads to the formation of endocytic pits from which clathrin-independent carriers are generated for toxin trafficking to early endosomes. From there, the holotoxins are transported via the retrograde trafficking route to the endoplasmic reticulum (ER), via the Golgi apparatus. The catalytic A-subunit is then translocated to the cytosol where it inhibits protein biosynthesis by modifying ribosomal RNAs (not shown). Right: In STxB (green)-based vaccines, antigens (blue) are linked via covalent bonds to the vector. The endocytic process then operates as for Shiga holotoxin molecules. While STxB-antigen conjugates also undergo retrograde trafficking (not shown), a small fraction of them escapes from the lumen of endosomes to reach the cytosol (endosomal escape). Here, proteasomes process the antigens to generate antigenic peptides, that are then imported into the lumen of the ER (or of endo/phagosomal processing compartments; not shown) for loading onto MHC class I molecules and subsequent presentation at the plasma membrane to CD8^+^ T cells.

The bacterial Shiga toxin belongs to the family of AB5 toxins [1]. These are composed of a catalytic A-subunit and a homopentameric B-subunit which is made from five identical B-fragments. The B-subunits of AB5 toxins bind to glycans of cellular protein or lipids [2]. The cellular receptor of the B-subunit of Shiga toxin (abbreviated as STxB) is the glycosphingolipid globotriaosylceramide (Gb3 or CD77) [3]. Of note, STxB is needed not only for toxin binding to cells, but also for the trafficking of the catalytic A-subunits inside the cells [4] (Figure 1). With the help of STxB, the A-subunit of Shiga toxin is delivered into the cytosol where it inhibits protein biosynthesis by modifying ribosomal RNA. This leads to cell death and contributes to the overall pathology that is associated with Shiga toxin producing enterohemorrhagic *Escherichia coli* bacteria, which bring about hemolytic-uremic syndrome, the leading cause of pediatric renal failure [5,6], but which also poses health risks to adults [7].

The endocytic and intracellular trafficking of STxB has been analyzed in some detail (Figure 1). At the plasma membrane, STxB reorganizes lipids, including its receptor glycolipid Gb3, in a way such that narrow tubular endocytic pits are formed [8] by exploiting a specific geometry of Gb3 binding sites on STxB [9] and its capacity to induce an asymmetric compressive stress onto the membrane leaflet to which it binds [10]. STxB-Gb3 complexes are then clustered by membrane-mediated mechanism, likely involving lipid fluctuation forces [11]. This mechanism of building endocytic pits without the need for the conventional clathrin machinery has been termed the glycolipid-lectin (GL-Lect) hypothesis [12,13]. This GL-Lect mechanism has been suggested to apply also for the structurally related glycolipid-binding B-subunit of cholera toxin [14,15,16].

The toxin-induced tubular endocytic pits then detach by scission from the plasma membrane to form clathrin-independent endocytic carriers [17]. This scission reaction involves the conventional pinchase dynamin [8] and also actin-driven domain boundary forces [18] and a mechanism that has been termed friction-driven scission in which the pulling of the molecular motor dynein on tubular endocytic pits that are scaffolded by the BAR domain protein endophilin leads to the thinning of their necks and to subsequent detachment [19,20]. The thereby generated clathrin-independent endocytic carriers are then targeted in a SNARE protein-dependent manner to early endosomes [21].

From early endosomes, STxB is delivered by retrograde transport to the endoplasmic reticulum, via the Golgi apparatus (reviewed in [22,23,24]) (Figure 1). From there, the catalytic A-subunit is translocated to the cytosol using the cellular retrotranslocation machinery [25].

## 2. Gb3 Expression and Membrane Translocation of STxB

In the healthy organism, the Gb3 glycolipid is found to be expressed in a limited number of tissues. In agreement with the fact that renal pathology is the most striking clinical manifestation that is associated with Shiga toxin, cells of microvascular glomeruli and proximal tubules have high Gb3 levels [26,27]. Gb3 is also found on microvascular endothelial cells, and in individuals who are infected with Shiga toxin producing *E. coli* strains, vascular endothelia of colon and brain are affected [28,29,30]. Platelets and erythrocytes also express Gb3 [31,32], as much as some immune cells such as germinal center B lymphocytes [33], monocytes, macrophages, and dendritic cells (DCs) [34].

DCs are key cells for the induction of primary immune responses, and notably also of CD8^+^ cytotoxic T lymphocytes (CTL) through the cytosolic processing and the cross-presentation of exogenous antigens (see below). It was therefore of interest when it was found that STxB (in some studies covalently coupled to cargo proteins such as antigens), in addition to reaching the retrograde trafficking route, also has the propensity to escape from the lumen of endosomes to reach the cytosolic compartment of DCs [34,35,36] (Figure 1). Using a quantitative assay, it was measured that roughly 0.5% of cell-associated STxB was translocated to the cytosol within 4 h incubation at 37 °C [37], which is in the range of the numbers that were described for other delivery systems [38]. The exact mechanism underlying this endosomal escape capacity remains very little understood.

## 3. Targeting of DCs: A Competitive Approach for Vaccine Development

### 3.1. A Brief History

Today’s marketed preventive vaccines induce antibodies that block different pathogens from infecting host cells. Prior to the success of RNA vaccines, most of these prophylactic vaccines were based on the administration of recombinant proteins. In other clinical situations such as chronic infections or cancer, other immune effectors such as CD8^+^ T lymphocytes must be mobilized for the development of therapeutic vaccines. At the end of the 1990s, apart from live attenuated vaccines and recombinant viruses which pose safety issues, there were no inactivated vaccines capable of inducing CD8^+^ T cells. Indeed, the recombinant proteins used for immunization were internalized in the endosomal pathway and processed peptides were presented to CD4^+^ T lymphocytes activating a humoral response. Different groups have therefore become interested in toxins because of their capacity to undergo endocytic trafficking and to deliver their catalytic subunits into the cytosol [39,40]. Access to the cytosol is indeed a prerequisite for the targeting of exogenous antigens into the HLA class I-restricted presentation pathway (cross-presentation) for their recognition by CD8^+^ T cells.

The teams of Claude Leclerc and Daniel Ladant were the first to show that a recombinant toxin derived from adenylate cyclase A produced by the bacterium *Bordetella pertussis* and incorporating a peptide derived from the lymphocytic choriomeningitis virus (LCMV) nucleoprotein triggered antigen cross-presentation to CD8^+^ T cells [41]. These teams also demonstrated that administration of recombinant cyclase A with a model peptide antigen was able to induce CD8^+^ T cells in mice [42]. Similarly, Pseudomonas exotoxin and anthrax toxin were then also shown to target antigens into the MHC class I presentation pathway [43] and to induce cytotoxic CD8^+^ T cells in mice [44,45,46,47].

In 1998, our teams published a first study showing that also the non-toxic STxB could be used to target antigens to the MHC class I-restricted pathway in human mononuclear cells and DCs [48]. Importantly, this presentation was shown to follow the proteasomal and transporter associated with antigen processing (TAP)-dependent pathway ensuring that with this vaccine strategy naturally occurring peptides are presented [34] (Figure 1).

Professional antigen presenting cells such as DCs express costimulatory molecules [49]. When cells that do not express these costimulatory molecules present HLA class I-restricted antigens to CD8^+^ T cells, this often leads to tolerance [50]. It was therefore of primary importance when we showed that Gb3 is preferentially expressed by DCs, and that STxB functions as a delivery tool for the in vivo targeting of antigens to DCs and for the improvement of immune responses, in particular by CD8^+^ T cells [34]. Consistently, we also demonstrated the role of DCs in the vaccine function of STxB [51]. 

### 3.2. Which DCs and Which Receptors to Target

In mice, DCs include DC1 (also known as Batf3-dependent, CD103^+^ tissue resident and CD8α^+^ lymphoid resident DCs), myeloid CD8α negative CD11b^+^DC2, and plasmacytoid DCs (B220^+^, Bst2^+^ and SiglecH^+^). In humans, a correspondence with this classification has been found with DC1 defined as CD141(BDCA-3)^+^ Clec9A (DNGR1)^+^CXR1^+^ DCs, DC2 identified as CD1c/BDCA-1^+^ DCs, and plasmacytoid DCs expressing BDCA2 (CD303^+^). For the skin, Langerhans cells and dermal DCs (CD14^+^) need to be added to this listing [52,53].

Initial studies showed that CD8α^+^DC in mice and BDCA3^+^ in humans had a higher cross-presentation capacity than the other subpopulations, thereby explaining their ability to preferentially induce CD8^+^ T cells [54,55]. On the contrary, CD8α negative DCs and Langerhans cells preferentially induce humoral responses [56]. Targeting DC2 via DCIR2 in mice [56,57] and Langerhans cells via langerin was more effective in activating follicular helper T cells and a humoral response than targeting DC1 [58,59].

**Figure 2:**
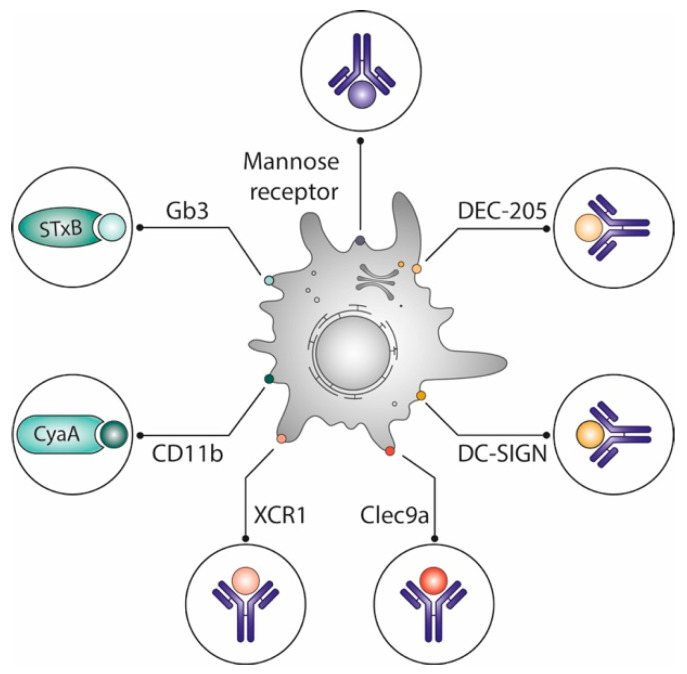
Optimization of vaccines by delivering antigens to dendritic cells. Different vaccine delivery systems preferentially target antigens to dendritic cells, which are known for their capacity to prime naive T cells: Vectors derived from toxin subunits such as the non-toxic STxB, which binds to the glycosphingolipid Gb3, or adenylate cyclase A, which binds to CD11b; antibodies targeting lectins (DEC-205, DC Sign) or other surface markers (mannose receptor, XCR1, Clec9a) of which some are specifically expressed on DC subpopulations. See text for details.

In the light of these findings, targeting highly specific DC1 markers such as Clec9a and XCR1 appeared as the most promising strategy for CD8^+^ T-cell induction (Figure 2). However, converging evidence has since accumulated that has challenged this idea on a functional dichotomy of DCs. It was indeed shown that all DCs are capable of cross-presentation in humans, especially when antigens are targeted to early endosomes [60,61,62].

In mice, STxB does not specifically target only CD8α^+^DCs [51], and adenylate cyclase A binds to the myeloid DC-specific CD11b [63]. Yet, both vectors potently induce CD8^+^ T cells. Other studies have also shown that engagement of different DC subpopulations as observed with STxB and other vectors is more effective for the induction of potent immune responses than targeting single DC subpopulations [64,65,66].

For the development of immunotherapy strategies, the structural homology and similarity of tissue expression patterns between humans and mice of the targeted DC surface markers also are important criteria to consider. For example, while in humans and mice DEC-205 is expressed at relatively high levels on myeloid blood DCs, the protein is, only in humans, also present on monocytes, B lymphocytes, NK cells, plasmacytoid blood DCs, and T lymphocytes [67,68,69]. Another example is the DC inhibitory receptor 2 (DCIR2, 33D1, Clec4A, CD367), which in mice is exclusively expressed by CD8-negative resident splenic DCs [56,70,71,72], while the protein is found on all blood DC subsets, monocytes, and granulocytes in humans [70,73,74]. It is therefore of note that the globotriose glycan to which STxB binds is structurally the same in all species, and that according to the current state of knowledge the expression pattern of the Gb3 glycolipid on DC populations is similar between species.

Considerations of differences between species also apply in the choice of adjuvants. For example, it has been reported that TLR9 is found on all major murine DC subsets, while the protein is only expressed by pDC in humans [75].

### 3.3. DC Maturation and Role of Adjuvants

Among the DC targeting vectors, some are known to induce DC maturation (e.g., anti-CD40, adenylate cyclase A, anti-Dectin-1) [76,77,78], while others do not (e.g., anti-DEC-205, anti-CD11c, anti-Clec9a, anti-Siglec H) [79,80,81,82]. When used without adjuvants or associated inflammatory stimuli, some of the latter, such as anti-DEC-205, even induce tolerance rather than activation of immune cells [80,81]. With anti-DEC-205, this has been used in mouse models to prevent the onset of diabetes [83] or of experimental autoimmune arthritis [84]. An anti-Siglec H antibody coupled to a Mog peptide similarly inhibits T-cell dependent autoimmune reactions in a murine EAE model when it is administered without adjuvant [85].

Other vectors like adenylate cyclase A that trigger DC maturation via the TLR4/TRIF pathway [77] induce CD8^+^ T cells without adjuvants. Vectors capable of inducing DC maturation, e.g., anti-CD40 or anti-dectin-1, have themselves been used as vaccine adjuvants [86,87].

One group reported a role for STxB in the maturation of DCs in the spleen and nasal-associated lymphoid tissue (NALT) [88,89]. However, this has not been seen by another team [90], and we did not observe any effect of endotoxin-free STxB on DC maturation [51]. Furthermore, we found that antigens must be conjugated to STxB for the induction of immune responses in mice [34]. If antigens and STxB are co-administrated, no immunomodulation is observed [34].

Unlike for anti-DEC-205, STxB-antigen conjugates induce cellular and humoral immune responses without the need for adjuvants [34,51,91]. Adjuvants such as αGalCer, CpG or poly(I:C) (a TLR3 ligand) nevertheless significantly improve specific CD8^+^ T-cell responses against antigens that are delivered via STxB, as one would expect for a delivery tool that does not itself activate DCs [92].

From a clinical perspective, the optimal combination of these vectors with adjuvants will result in specific immune response profiles. Thus, antigenic targeting by anti-Clec9A without associated adjuvant promotes proliferation and induction of antigen-specific regulatory CD4^+^ T cells, while coadministration of poly(I:C) or curdlan (Dectin-1 ligand) promotes the generation of antigen-specific Th1 or Th17 cells, respectively [93].

### 3.4. Systemic Immune Responses Induced by STxB and Other DC Targeting Vectors

DC targeting vectors are developed for the induction of CD8^+^ T cells (Figure 2). Indeed, many vectors (anti-CD40, anti-Clec9a, anti-DEC-205, adenylate cyclase A, anti-Dectin-1, anti-mannose receptor...), when coupled to different antigens have been shown to be more efficient than the non-vectorized antigens for the generation of CD8^+^ T cells in mouse models [78,94,95,96,97,98,99,100,101]. Some of these vectors (i.e., anti-CD40, anti-Clec9a, anti-Dec-205) have even been shown to induce CD8^+^ T cell in non-human primates [102,103,104,105,106].

In wild-type mice of different genetic backgrounds and in humanized mice, conjugates of STxB with antigenic peptides or proteins induce antigen-specific CD8^+^ T cells, which are detectable ex vivo and persistent over time [34,51,91,92,107]. Of note, only a few micrograms of STxB-antigen vaccine are required per mouse to induce CD8^+^ T cells when adjuvants are used, as opposed to the situation with non-vectorized antigens [92]. Similar observations have been reported for strategies that are based on targeting DEC-205 or XCR1 [97,108]. 

Most of these vectors also drive the presentation of antigen-derived peptides by HLA class II molecules to CD4^+^ T cells, thereby inducing antibodies with titers that are higher when compared to those obtained by vaccination with non-vectorized antigens. Some vectors (STxB, anti-XCR1, adenylate cyclase A) cause TH1 polarization with the production of IgG2a isotype antibodies [91,99,109], while others (anti-DEC-205, anti-Dectin 1) result in a mixed TH1/TH17 polarization [110,111,112]. Some vectors (anti-Lox-1, anti-Clec9a, anti-DCIR2) appear to be particularly effective in promoting antibody induction, which might be linked to their ability to activate follicular CD4^+^ T cell [103,113,114,115]. These mixed humoral and cellular responses provide additional arguments to suggest that the attempt to match the targeting of given types of DC with specific types of immune responses might be an oversimplification.

### 3.5. Protection against Viral Infection and Tumor Growth

In different preclinical models of wild-type or humanized mice it was shown that systemic administration of STxB-antigen conjugates inhibits tumor growth both in the context of prophylactic or therapeutic vaccination [51,107,116]. In most of these models, protection was conferred by CD8^+^ T cells. STxB-antigen conjugates also protect against infection caused by a smallpox-derived virus [92] or against bacterial infection caused by *Boretella pertussis* [117]. Interestingly, coupling of a STxB-related toxin, STx2b, to a clostridium perfringens-derived enterotoxin enhances the humoral response against enterotoxin and provides protection against this pathogenic product [118]. It was also found that vectors targeting DEC-205, Clec9a, XCR1, CD11b (via adenylate cyclase A), or CD40 conferred protection against infectious disease [109,119,120,121,122,123] and tumor growth [96,97,98,124,125,126,127,128,129,130].

## 4. STxB Functions as a Mucosal Delivery Vector 

### 4.1. The Mucosal Immune System and Its Specific Effectors

The mucosal immune system, also called MALT (mucosa-associated lymphoid tissue), is an integrated and well-organized architecture covering the lung, head and neck, digestive and genital mucosa. It is made of lymphoid follicles that are associated with a layer of T, B, and antigen presenting cells. These immune cells, which are close to the epithelium and M cells, represent between 10 and 20% of the epithelial barrier. M cells play an important role in the internalization and transfer of antigens to DCs [131]. A first priming of immune responses takes place at this follicle-associated epithelium (FAE), which is also called mucosal inductive site. Thereby induced immune cells reach the adjacent lymph nodes upon which they return to the very mucosa in which they had been generated [132,133]. 

Compared to the immune response induced by peripheral lymph node priming, the mucosal immune response is characterized by two immune effectors that are specifically found in mucosal tissues: secretory IgA and resident memory T cells [134]. In contrast to IgA in serum, secretory (s)-IgA antibodies are produced locally in the mucosa and are more resistant to bacterial enzymes. sIgAs are the key immune effector molecules in the mucosa. After binding to polymeric immunoglobulin (Ig) receptors (pIgRs), sIgAs are transported across mucosal epithelial cells to the intestinal lumen or other mucosa. Only mucosal and not systemic immunization pathways can generate them [135,136]. Their presence in mucosal sites is associated with optimal vaccine protection against viral infections [137,138].

More recently, a new non-recirculating lineage of T cells has been described in mucosal tissues, which were termed tissue resident memory (T_RM_) T cells [139,140]. T_RM_ specifically differentiate in the mucosal tissue and are not found in the blood. They express the CD103 marker which binds to epithelial cell-specific E-cadherin. T_RM_ are thought to play an immunosurveillance role in the mucosa. Their presence in the vicinity of the epithelium allows them to act rapidly in the event of infection and to promote the swift recruitment of new effectors without the need for a lengthy T-cell differentiation process in lymph nodes [141]. T_RM_ have also been found in tumors especially in mucosal localization and are associated with a favorable prognosis [142,143]. As for sIgAs, mucosal routes of immunization are more efficient in inducing T_RM_ than conventional systemic routes [144].

### 4.2. STxB—The First Non-Live Mucosal Delivery Vector That Induces T_RM_

We showed that intranasal immunization with conjugates between STxB and the E7 protein from human papilloma virus 16 (HPV16) is more effective in inducing mucosal IgAs and anti-E7 CD8^+^ T cells in the lung than intramuscular immunization [145]. Intranasal STxB-E7 immunization promotes intratumoral CD8^+^ T cell recruitment and the regression of E7-expressing tumor in the lung or head and neck mucosa. In contrast, intramuscular immunization with STxB-E7 induces CD8^+^ T cells in blood and spleen, but not in the lung and has no significant effect on the growth of a tumor xenograft in the tongue. The intranasally induced CD8^+^ T cells express CD103 and CD49a and have a T_RM_ phenotype. Of note, these cells are not induced when STxB-E7 is injected via the intramuscular route of immunization [145,146].

In a series of experiments based on the elimination of T_RM_, the blocking of their differentiation or migration, or their isolation by parabiosis, we have clearly shown their role in the inhibition of tumor growth after immunization of mice with different STxB-antigen conjugates [146]. More recently, we have shown that T_RM_ preferentially express the chemokine receptor CXCR6, when compared to effector CD8^+^ T cells [147]. Immunization via the intranasal route and not the intramuscular route allows to induce the chemokine CXCL16 in the lung, which could explain the recruitment of T_RM_ [147].

These studies demonstrate for the first time that a protein-based vector targeting DCs induces T_RM_, and that the nasal immunization route is required for this. Earlier work had already pointed to the possibility that STxB might act as a mucosal delivery vector. Indeed, a STxB fusion protein with a rotavirus NS4 polypeptide was shown to increase intestinal IgA concentrations and serum IgG when administered orally, and to protect breastfeeding pups against diarrhea after an infectious challenge [148].

### 4.3. Other Mucosal Vaccination Strategies

Preparations based on vesicular stomatitis virus (VSV), adenovirus 26 (ADV26), or modified vaccinia virus Ankara have enabled the commercialization of vaccines against Ebola virus [149,150]. Intranasal administration of recombinant preparations based on cytomegalovirus (CMV)-derived viruses, influenza virus, ADV, VSV have been shown to induce IgA and T_RM_ in different mucosal locations [151,152,153,154]. In a preclinical model of infection with SARS-CoV-2, a recombinant chimpanzee ADV (ChAdOx1)-encoding SARS-CoV-2 Spike administered nasally or subcutaneously was shown to protect against lung infection after a viral challenge, but only intranasal administration of the vaccine protects against upper airway infection. This protection is associated with the preferential induction of local mucosal IgA and T_RM_ [155].

Few non-live vectors have been tested for their ability to deliver antigens via the mucosal route. For example, conjugates between the non-toxic B-subunit of cholera toxin and bacterial or viral antigens increase antigen-specific IgA compared to non-vectorized antigen when they are administered nasally or sublingually [156,157]. Upon nasal or subcutaneous administration, a scFv directed against DEC-205 and coupled to a parasite antigen increases IgA concentrations in nasal washings as well as a CD4^+^ T-cell response in the spleen, allowing partial protection against a parasite challenge [158].

More generally, two main mucosal delivery tools are developed for vaccine:

(i) Lactic acid bacteria (LAB) that include *Lactobacillus* spp., *Lactococcus* spp., and *Streptococcus* spp. LAB are generally recognized as safe and considered as transiting and non-invasive bacteria [159,160].
(ii) Nanoparticles, i.e., (a) polysaccharide-based natural polymers such as chitosan, pullulan, alginate, inulin, hyaluronic acid, maltodextrin; (b) lipid-based delivery systems (i.e., cationic liposomes, virions, archaeological bodies, small cochlea, and immunostimulating complexes); (c) synthetic polymeric nanoparticles (poly(lactic-co-glycolic acid), polycaprolactone, polyahydrides, polyphosphazene). These polymers have the advantage of being biodegradable.

After mucosal administration, LABs and nanoparticles generate mucosal responses against entrapped antigens [161,162,163,164]. To improve their efficacy, LABs such as lactobacillus have been coupled with DC targeting peptides; alternatively, complement C3d3, anti-CD205, anti-CD11c, or neonatal Fc receptors (FcRn) have been expressed at their surface [165,166,167,168]. Nanoparticles such as poly(lactic-co-glycolic acid) and liposomes have also been functionalized with anti-DEC-205 [169], anti-CD40 [170], anti-mannose receptor [171], or anti-CD11c [172] antibodies to target them to DCs. These elegant strategies, which combine mucosal delivery, DC targeting, and the possibility to incorporate multiple cargo molecules are up until now limited by issues related to reproducibility of their synthesis and scale up for clinical application.

Regarding RNA vaccines, their direct intranasal administration without encapsulation does not lead to the induction of a mucosal immune response [173]. Some studies show that their encapsulation as nanoparticles, cationic liposome/protamine complexes (LPC), or mannose-conjugated lipid nanoparticles generate cellular responses that inhibit tumor growth [173,174,175]. Xun Sun’s group demonstrated that cationic cyclodextrin-polyethylenimine 2k conjugates (CP 2k) which are complexed with anionic mRNA-encoding HIV gp120 induce strong systemic and mucosal anti-HIV immune responses [176]. Nevertheless, toxicity problems have been reported with polyethyleneimine and lipid nanoparticles when these are injected via the nasal route [177,178,179]. Improving the benefit-risk balance and the efficacy of these mucosal RNA vaccines is the subject of numerous ongoing studies.

## 5. STxB in Combination with Other Cancer Treatment Modalities

Apart from a few positive clinical signals of therapeutic HPV vaccines in pre-neoplastic cervical lesions, no therapeutic vaccine has demonstrated sufficient efficacy in patients with advanced cancer or chronic infection (e.g., HIV) to change clinical practice [140]. An in-depth investigation of the tumor microenvironment has revealed the existence of immunosuppressive mechanisms that likely explain the failure of therapeutic vaccines in advanced stage cancers [180]. Indeed, T cells that migrate into tumors quickly become exhausted and express inhibitory receptors like PD-1. Blocking the interaction between PD-1 and PD-L1 has led to the success of immunotherapy in many clinical indications [181,182]. Second generation immunotherapy protocols are therefore developed in which an inhibition of the PD-1/PD-L1 pathway is combined with vaccines or conventional treatments [183]. 

In preclinical models, we were one of the first teams to show that this combinatorial approach might indeed be successful [184]. In mice with HPV E7-expressing tumors, administration of either a STxB-E7 vaccine or an anti-PD-1 antibody led to only a partial therapeutic response. In contrast, the combination of both induced total tumor regression [184]. The value of combining a STxB-based vaccine with anti-PD-1 antibodies (and the local injection of IFNα) was also confirmed by another group [185,186].

Regulatory T cells are another type of the immunosuppressive cells in the tumor microenvironment that counteract vaccine efficacy. We have shown that the combination of a Treg inhibitor targeting the CCR4 pathway with a vaccine composed of STxB coupled to self-antigens overcomes tolerance and allows to eliminate tumors that express these self-antigens [187]. This combination proved to be effective in inhibiting the growth of numerous tumors (i.e., melanoma, colon cancer, and lung cancer). A similar synergistic effect was observed in the presence of a mTor pathway inhibitor [188].

In many clinical indications, a therapeutic vaccine would need to be combined with conventional treatments such as radiotherapy or chemotherapy. In collaboration with Eric Deutsch’s group, we have shown in a head and neck cancer model that radiotherapy increases the effect of a STxB-E7 vaccine by making endothelial cells more permissive to infiltration by CD8^+^ T cells [189].

As summarized above and also in other studies [190,191], the STxB vector has been used reproducibly by independent groups for the preclinical development of immunotherapy applications. These studies support the design of clinical trials including STxB-based vaccines in 2nd generation immunotherapy strategies.

## 6. Potential Limitations of STxB

### 6.1. Intrinsic Immunogenicity and Toxicity

One of the potential problems with the use of a vector derived from an exogenous protein is the presence of pre-existing antibodies or the development of a neutralizing immune response against the vector. For STxB, we have addressed these aspects in mice. Upon immunization of mice with STxB-antigen conjugates, an antibody response is observed against STxB, which is 100-fold lower than the one directed against the antigen itself, however [92]. Moreover, anti-STxB antibodies do not interfere with the induction of a CD8^+^ T-cell response against the antigen. Indeed, the intensity of the CD8^+^ T response increases in the same animal with repetitive immunizations, and if an animal is pre-immunized with non-antigen-coupled STxB at high doses, the CD8^+^ T-cell response is not diminished upon vaccination with STxB-antigen conjugates, compared to mice that were not pre-immunized [92].

The low immunogenicity of STxB is also observed in humans. Indeed, in serum samples from 30 patients with hemolytic-uremic syndrome (HUS), caused by Shiga toxin producing *E. coli* strain O157:H7, no antibodies against the toxin could be detected [192]. In other studies, on clinical samples, antibodies against Shiga toxin were present, but not against STxB, or only with a very low frequency of 1.3% [193,194]. We also detected the presence of anti-STxB antibodies in only 2 out of 30 serum samples of patients with HUS. Furthermore, our teams also did not find antibodies against STxB in sera of healthy subjects [192], and the frequency of antibodies directed against holotoxin was found to be about 1.8% in healthy subjects [195]. These studies demonstrate the low immunogenicity of STxB and the absence of pre-existing antibodies against this protein in humans. This low intrinsic immunogenicity gives STxB an important advantage over viral vectors.

Different approaches have been used to address a potential toxicity of STxB. Using a classical screening test for toxicity, i.e., the rabbit reticulocyte lysate system, no inhibition of globin synthesis was observed with up to 100 µg per reaction of Shiga toxin that by mutation was rendered devoid of enzymatic activity [196]. Mice treated intraperitoneally with toxoids of STx1 or STx2 whose catalytic sites were mutated at protein concentrations equivalent to more than 100 times the ones used for immunization showed no ill effects [197]. In another study, mice were given STxB doses as high as 200 mg/kg [198] or 220 µg per mouse [88]. Yet, no signs of clinical toxicity were observed, including mucosal sites. In our immunization experiments, mice were given three doses of up to 80 μg of STxB at 3-week intervals, which corresponds to 8 mg of STxB per kg of mouse weight. These mice did not exhibit any signs of clinical morbidity with a follow-up of 6 months (Ref. [51] and unpublished).

### 6.2. Production

For all studies that have been discussed above on STxB as an antigen delivery tool, the protein was purified from bacteria, which are its natural hosts. STxB is obtained in amounts that are typical for proteins which are expressed in the periplasmic space. STxB has recently also been chemically synthesized and refolded in vitro [199]. Whether this type of material can also be used to obtain functional vaccine conjugates remains to be tested.

**Figure 3:**
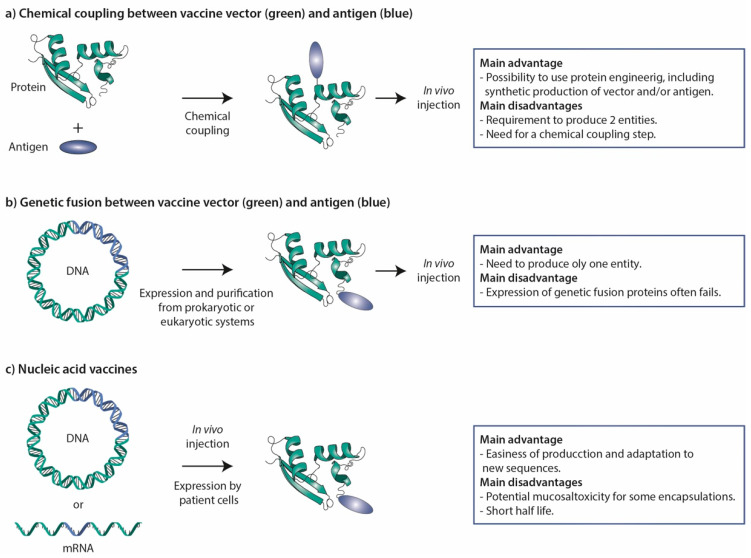
Vaccine strategies. (**a**) Chemical coupling. In this procedure, vector and antigen are produced in parallel and then chemically coupled to generate the vaccine. This approach has been extensively used for STxB. (**b**) Genetic fusion. Vector and antigen are genetically fused at the cDNA level, and then expressed in and purified from prokaryotic or eukaryotic cell systems. In some cases, this approach has been used for STxB, but often corresponding fusion proteins could not be obtained. (**c**) Nucleic acid vaccines. Fusion proteins between vectors and antigens are expressed from DNA or mRNA molecules that are directly injected into the organism. These vaccine molecules are thereby produced by the cells of the organism receiving the vaccine. The main advantages and disadvantages of the different strategies are listed to the right.

Antigenic peptides and proteins can in principle be genetically fused or chemically coupled to STxB (Figure 3). Even if successful in a few cases [48,148], genetic fusions in most cases fail to be found at significant levels in the bacterial periplasm. A chemical coupling approach was therefore favored in most studies (Figure 3).

For chemical crosslinking, a variant was designed in which a cysteine was added to the C-terminus of each B-fragment. It turns out that despite this supernumerary cysteine, the intrachain disulfide bond at the level of each B-fragment still forms with high specificity. This variant, termed STxB/Cys, is expressed and purified with similar efficiency as wild-type STxB, conserves the Gb3 binding and intracellular transport characteristics of the wild-type protein, and lacks toxicity. It has been conjugated to a large variety of molecular entities from fluorophores [200] and radioelements [201,202] to peptides [145], full-size proteins [36,91] and liposomes [203].

## 7. Conclusions

STxB appears to be a competitive DC-targeting vector for CD8^+^ T-cell induction, which remains a challenge in vaccinology. Other vectors such as adenylate cyclase A from *Bordetella pertussis* or anti-CD40 antibodies have also demonstrated their ability to induce CD8^+^ T cells. However, in a randomized phase II clinical trial no significant difference compared to placebo was observed in viral clearance in women with HPV16/18 cervical lesions vaccinated with the GTL001 vaccine composed of a recombinant HPV16–18 adenylate cyclase vaccine [204]. The use of anti-CD40 antibodies has been hampered by clinical toxicity [205,206]. Other recent data have positioned STxB as the first non-live mucosal vector capable of inducing mucosal IgA immunity and mucosal T_RM_, which play key roles in controlling pathogens and in anti-tumor immunosurveillance [145,146]. In contrast, other non-live vectors such as DEC205 ligands, nanoparticles, and mRNAs, when administered via the mucosal route, fail to induce mucosal immunity. Furthermore, the expression of DEC205 differs depending on species; it is less specific of cDC1 in humans, and ligands of DEC205 as delivery vectors induce tolerance [67,81]. Regarding functionalized nanoparticles, problems have been observed concerning the reproducibility of their synthesis and the feasibility of their industrial scale up, while mucosal administration of mRNA encapsulated in lipid nanoparticle appears to be toxic [173,177,207]. Finally, the low immunogenicity of STxB, its lack of toxicity, binding to a receptor that is conserved across species, and the reproducibility of vaccine efficacy that was obtained with this vector by different independent groups [91,185,186,190,191] make it an attractive antigen delivery tool, particularly for the development of mucosal vaccines in infectious diseases and cancer. Its potential use in the context of nucleic acid vaccines awaits further exploration (Figure 3).

## Data Availability

Not applicable.
